# Supplementary material for: The interactome of intact mitochondria by cross-linking mass spectrometry provides evidence for coexisting respiratory supercomplexes
Source: Mol Cell Proteomics. 2017 Dec 8;17(2):216–32. doi: 10.1074/mcp.RA117.000470 (PMC5795388; doi:10.1074/mcp.RA117.000470)
Supplement: Supplemental Data [file supp_RA117.000470_133922_0_supp_23978_fzffwf.zip › spectra_annotation/mito_spectra_annotation/305-1-2-1-4-1.pdf]

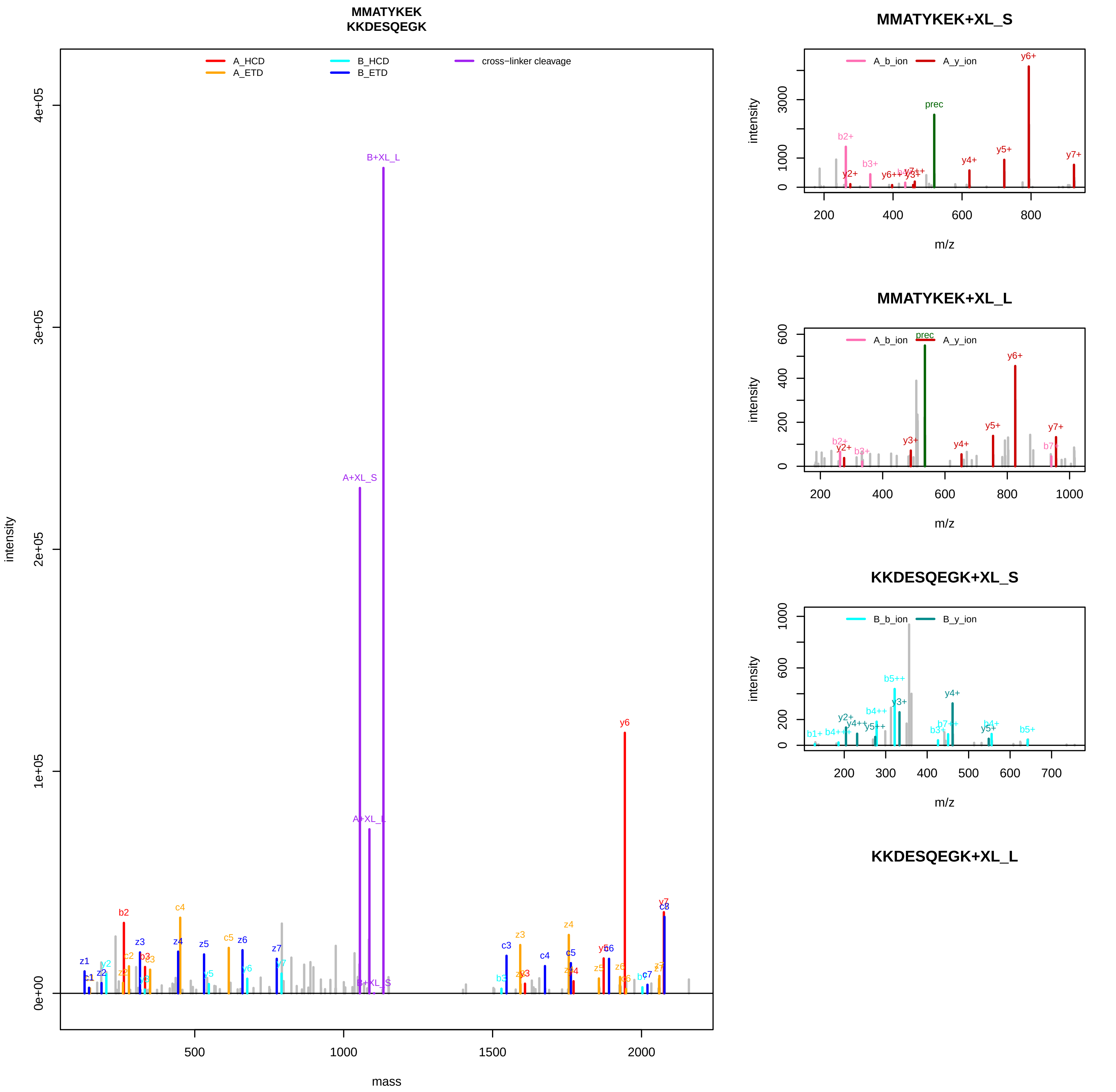
MMATYKEK
KKDESQEGK
A_HCD
A_ETD
B_HCD
B_ETD
cross−linker cleavage
intensity
mass
B+XL_L
A+XL_S
A+XL_L
B+XL_S
y6
y7
c8
b2
c4
z3
z4
z5
c5
z6
z7
c3
z3
c4
z4
c5
MMATYKEK+XL_S
A_b_ion
A_y_ion
prec
y6+
y5+
y4+
y7+
b2+
b3+
y2+
m/z
MMATYKEK+XL_L
KKDESQEGK+XL_S
B_b_ion
B_y_ion
b5++
y4+
y3+
y2+
b4++
b1+
y5+
b5+
KKDESQEGK+XL_L
